# Pharmacokinetics of buprenorphine in pregnant sheep after intravenous injection

**DOI:** 10.1002/prp2.726

**Published:** 2021-02-22

**Authors:** Henriikka Hakomäki, Hannu Kokki, Marko Lehtonen, Veli‐Pekka Ranta, Juha Räsänen, Hanna‐Marja Voipio, Merja Kokki

**Affiliations:** ^1^ School of Pharmacy University of Eastern Finland Kuopio Finland; ^2^ School of Medicine University of Eastern Finland Kuopio Finland; ^3^ Department of Obstetrics and Gynecology Helsinki University Hospital Helsinki Finland; ^4^ Department of Experimental Surgery Oulu Laboratory Animal Centre Oulu University Hospital and University of Oulu Oulu Finland; ^5^ Department of Anesthesia and Intensive Care Kuopio University Hospital Kuopio Finland

**Keywords:** buprenorphine, pharmacokinetics, pregnancy, sheep

## Abstract

Buprenorphine is a semi‐synthetic opioid, widely used in the maintenance treatment for opioid‐dependent pregnant women. Limited data exist on the pharmacokinetics of buprenorphine in pregnancy. We conducted a pharmacokinetic study to determine the pharmacokinetics of intravenous buprenorphine in pregnant sheep. Fourteen pregnant sheep in late gestation received 10 µg/kg of buprenorphine as an intravenous bolus injection. Plasma samples were collected up to 48 h after administration. Buprenorphine and its metabolite, norbuprenorphine, were quantified from plasma using a LC/MS/MS method, with lower limits of quantification of 0.01 µg/L and 0.04 µg/L for buprenorphine and norbuprenorphine, respectively. The pharmacokinetic parameters were calculated using noncompartmental analysis. The pharmacokinetic parameters, median (minimum−maximum), were C_max_ 4.31 µg/L (1.93–15.5), *AUC*_inf_ 2.89 h*µg/L (1.72–40.2), *CL* 3.39 L/h/kg (0.25–6.02), terminal t½ 1.75 h (1.07–31.0), V_ss_ 8.04 L/kg (1.05–49.3). Norbuprenorphine was undetected in all plasma samples. The median clearance in pregnant sheep was higher than previously reported for nonpregnant sheep and human (male) subjects. Our sensitive analytical method was able to detect long terminal half‐lives for six subjects, and a wide between‐subject variability in the study population.

Significance statement: Buprenorphine is widely used for the treatment of opioid use disorder in pregnancy. However, limited data exist on the pharmacokinetics of buprenorphine during pregnancy. As this type of study cannot be done in humans due to ethical reasons, we conducted a study in pregnant sheep. This study provides pharmacokinetic data on buprenorphine in pregnant sheep and helps us to understand the pharmacokinetics of the drug in humans.

## Graphical abstract

In the present pharmacokinetic study with 14 pregnant sheep we have shown that the buprenorphine systemic clearance in pregnant sheep is higher than previously reported for non‐pregnant sheep and human subjects, and that a sensitive analytical method and a long sampling period are the key elements of detecting the true elimination phase of buprenorphine.

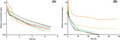

**Abbreviations**
AUC_%Extrap_: Percent of extrapolated AUC from AUC_inf_
AUC_inf_: Area under the curve from time zero to infinity
BUP: Buprenorphine
CL: Plasma clearance
C_max_: Maximum plasma concentration
IV: Intravenous
LC/MS/MS: Liquid chromatography ‐ tandem mass spectrometry
LLOQ: Lower limit of quantification
MRM: Multiple reaction monitoring
NBUP: Norbuprenorphine
t ½: Terminal half‐life
V_ss_: Apparent volume of distribution at steady‐state
V_z_: Apparent volume of distribution at the terminal phase

## 1 INTRODUCTION

Buprenorphine (BUP) is a partial agonist at µ‐opioid receptor and has antagonist effects at δ‐ and ĸ‐opioid receptors.^1^, ^2^ Agonistic interactions with the opioid receptor‐like 1 receptor could also contribute to the antinociceptive effect.^1^, ^3^ Due to high affinity and low dissociation rate from the opioid receptors, BUP is classified as a long‐acting opioid.^1^, ^4^ BUP is a small, highly lipophilic compound that is highly bound to plasma proteins (96%).^5^ BUP is metabolized in liver via CYP3A4 by N‐dealkylation into its main active metabolite, norbuprenorphine (NBUP). BUP and NBUP are further glucuronidated by uridine 5'‐diphospho‐glucuronosyltransferase (UGT) UGT1A1, UGT1A3, and UGT2B7 into BUP‐3‐glucuronide and NBUP‐3‐glucuronide. BUP and its metabolites are mainly eliminated through feces, and 10–30% of the administered dose is excreted into urine as water‐soluble metabolites.^6^, ^7^

Buprenorphine has been used for the treatment of moderate‐to‐severe pain and opioid use disorder since 1996. During pregnancy, BUP is not recommended for pain management but is commonly used in opioid substitution treatment for opioid‐addicted pregnant women. Despite the wide use, the pharmacokinetics of BUP during pregnancy is poorly understood.^7^

Several physiological and body composition changes during pregnancy could affect the pharmacokinetic of BUP. These include increased cardiac output, plasma volume, total body water and glomerular filtration rate, changes in the expression and activity of metabolizing enzymes (CYP3A4 and UGTs), and decreased protein binding.^8^ After sublingual administration, BUP exposure (area under the plasma concentration curve, AUC) is lower during pregnancy than postpartum in women receiving BUP for substitution treatment of opioid dependence.^9^, ^10^ In humans, BUP metabolic ratios (AUC of NBUP and NBUP‐glucuronide to the AUC of BUP) are higher during pregnancy compared with postpartum period.^11^ These findings may indicate an increased systemic clearance of BUP in pregnancy.

However, the pharmacokinetic data on BUP during pregnancy are sparse. In this study, we have determined the basic pharmacokinetic parameters of BUP in pregnant sheep after intravenous (IV) administration, for future reference in consecutive studies on BUP central nervous system permeation in the sheep model, and to eventually help us understand BUP pharmacokinetics in humans during pregnancy. We used a pregnant sheep model, as this type of study could not be conducted in humans, due to ethical reasons. In this study, 14 sheep were in the late stages of their pregnancies. Sheep was chosen as the animal model for the study, since the resemblance in size and weight is close to that of humans. Also, even though the gestational period is half of that of humans, the sheep fetus is of similar size and weight in late gestation as the human fetus. The size of the animal makes cannulation easy and enables sufficient blood samples to be collected from the animal. Another advantage with sheep is that they adapt to the laboratory environment and to handling fast and show little to no signs of stress during acclimation and experimentation. These features offer significant advantages in obstetric studies compared to rodent species. However, no animal model is exactly similar to humans. Even though the basic function of the placenta (e.g., hormone secretion, and transfer of nutrients and drugs) and hormonal profiles (e.g., estrogens and progesterone) are similar to all mammals during pregnancy, the placental interface differs between species and can affect the pharmacokinetics of a drug. In sheep, the placenta has one layer of maternal uterine endothelium and one layer of trophoblasts that separate the maternal and fetal circulation (epitheliochorial placenta), whereas, in humans, the maternal blood is separated from the fetal circulation with only one layer of trophoblasts (hemomonochorial placenta).^12^

To our knowledge, IV pharmacokinetics of BUP have not been reported in pregnant sheep. Pharmacokinetics of IV BUP have been investigated in nonpregnant sheep, but the studies have limitations, most importantly, a short study period. In Nolan and associates’ study with six adult female sheep, plasma samples were collected only up to 6 h after IV injection of 6 µg/kg BUP and in Lindhardt et al. three sheep were sampled for 1 h.^13^, ^14^ Due to the short sampling period, neither study was able to capture the true elimination phases of BUP.

The aim of our study was to quantify BUP and NBUP concentrations after a single IV bolus injection in pregnant sheep. Plasma samples were collected up to 48 h after administration, and BUP and NBUP were quantified with a highly sensitive LC/MS/MS method. Noncompartmental analysis was conducted to determine the individual pharmacokinetic parameters. Our study hypothesis was that pregnancy increases the systemic clearance (CL) of BUP compared to that reported for nonpregnant sheep.

## 2 MATERIALS AND METHODS

### 2.1 Animals

The animal transport, husbandry, and experimental procedures were carried out according to the Finnish national legislation and the EU directive 2010/63/EU.^15^, ^16^ The study protocol was approved by the National Animal Experiment Board of Finland (reference no. ESAVI/7840/04.10.07/2017).

The study was conducted on 14 time‐mated pregnant, 1‐ to 5‐year‐old (median 2) Åland landrace sheep (Lammastila Sikka Talu, University of Turku, Rymättylä, Finland). At the beginning of the study, the sheep weighted 48–77 kg (median 57 kg) being at 112–120 gestational days (median 115 days). Thus, all sheep were near term at the time of the study (term being 145 gestational days) and had 2–3 fetuses each.

The sheep were transported to the Laboratory Animal Center (Oulu, Finland) 2 weeks prior to the study for housing and acclimation. During the adaptation period, the sheep were group housed in two pens of 10.8 m^2^ in area and during the experiment in individual pens of 3.6 m^2^, with straw bedding. The room temperature was 18 ± 2°C, ventilation rate 15 times/h, and humidity 45 ± 5%, with a light‐dark cycle of 12:12 h. The sheep were given tap water and hay ad libitum and they had a salt block in the pen. Oat grains, turnip‐rape‐based protein supplement (Farmarin rypsi, Hankkija‐Maatalous Oy) and mineral and vitamin supplement (Lammas Hertta, Hankkija‐Maatalous Oy) were individually rationed and given twice daily. The rations were increased gradually toward the end of the pregnancy. When needed, supportive doses of calcium were given either orally or IV.

Animals were monitored throughout the study by veterinarians, animal technicians and the research team for signs of distress, pain, injuries, or diseases. Actions were taken immediately to improve the well‐being of the animals when needed.

### 2.2 Buprenorphine administration and sample collection

Prior to the drug administration, sheep's both external jugular veins were cannulated. The area was shaved, cleaned with soap, and disinfected with ethanol solution before cannulation. The right‐side jugular vein cannula was used for blood sampling and the left side for BUP administration.

Buprenorphine (Vetergesic vet 0.3 mg/ml; Ceva Santé Animale) dose of 10 µg/kg BUP‐free base was diluted in 10 ml 0.9% saline (sodium chloride) and given IV as a 1‐min injection through the left side cannula. The dose was based on a clinically administered dose for sheep.^17^ The BUP dose was well tolerated in all animals.

Blood samples, 4 ml, were collected prior to the BUP administration and then, at 5, 15, 30, 45, and 60 min, and at 2, 4, 7, 10, 24, 30, and 48 h after the IV administration. After blood sampling, the jugular vein cannula was flushed with 20 ml 0.9% NaCl and then with 2 ml 50 IU/ml heparin solution. The blood samples were collected in heparinized plasma tubes and centrifuged at 2000*g* for 10 min. Plasma was divided into two cryotubes and stored first at −35°C and then moved to −85°C until analysis.

### 2.3 Buprenorphine quantification

The plasma concentrations are expressed as BUP‐free base. Plasma samples were analyzed with quantitative liquid chromatography triple quadrupole mass spectrometric method (LC/MS/MS). This method was based on previously published methods,^18^, ^19^, ^20^ and adjusted and validated for sheep plasma analysis. Briefly, prior to analysis, BUP was isolated from samples by liquid–liquid extraction with toluene. The samples were kept on ice while they were being processed. The sample (100 µl of plasma) was transferred to a screw‐capped glass test tube and 5 µl of an internal standard solution (d4‐BUP, 10 µg/L in methanol) was added to the sample. 500 µl of toluene was added to the sample and samples were Vortex mixed for 30 s, further mixed for 5 min in Heidolph Multi Reax shaker (speed value 10), and then centrifuged at 290 *g* for 5 min at 10°C to achieve a sharp phase separation. The sample was incubated on dry ice for 5 min and the upper toluene layer was transferred to another screw‐capped glass test tube. The liquid extraction was repeated once with 250 µl of toluene, and the upper toluene layer was combined with the first toluene extraction. The sample was then evaporated to the dryness under nitrogen at 30°C and the residue was reconstituted in 100 µl of methanol‐water solution (2:1, v/v). The sample was allowed to dissolve for 5 min and was then transferred to a HPLC sample vial.

LC/MS/MS experiments were performed using an Agilent 1290 Series UHPLC System (Agilent Technologies) coupled to an Agilent 6495 Triple Quadrupole LC/MS with Jet Stream and iFunnel technology (Agilent Technologies). Five microlitre of plasma sample was injected onto a reversed phase HPLC column (Kinetex 1.3 µm C18, 50 × 2.1 mm, Phenomenex). The column temperature was 60°C, flow rate 0.4 ml/min, and gradient elution was used with water (eluent A) and methanol (eluent B), both containing 0.05% (v/v) of formic acid. The following gradient profile was employed: 0–0.5 min: 15% B; 0.5–5.0 min: 15 → 95% B; 5.0 → 7.0 min: 95% B; 7.0 → 7.1: 95 → 15% B; and 7.1–9.0 min: 15% B. The sample tray was maintained at 10°C. A Jetstream ESI (electrospray ionization) conditions in positive ion mode consisted of a source temperature of 180°C, drying gas flow of 15 L/min, capillary voltage 3000 V, a nebulizer pressure of 40 psi, a sheath gas flow of 11 L/min, and a temperature of 400°C. The nitrogen was used as the instrument gas. Detection was performed using multiple reaction monitoring (MRM) with a dwell time of 50 ms and fragmentor voltage of 380 V. Collision energy values of 30 and 54 V were used for the analysis of BUP and d4‐BUP, respectively. The following MRM transitions were used: *m*/*z* 468 → 468 and *m*/*z* 468 → 55.2 for BUP, *m*/*z* 414 → 414 NBUP, and *m*/*z* 472 → 472 and *m*/*z* 472 → 59.2 for d4‐BUP. Mass resolution for MS1 and MS2 quadrupoles was 0.7 FWHM and 1.2 FWHM in the analysis of plasma samples, respectively. The lower limit of quantification (LLOQ) in plasma samples for BUP was 0.01 µg/L and 0.04 µg/L for NBUP with an upper limit of linearity of 25.0 µg/L. The intra‐ and inter‐day (five replicate samples each day at 0.078, 0.313, and 2.5 µg/L, on three separate days) accuracy (%Bias) and precision (%Coefficient of variance) were below 16% for BUP and below 25% for NBUP. The mean recoveries were 84–102% for BUP and 78–105% for NBUP, for the tested concentrations.

### 2.4 Data analysis

The pharmacokinetic parameters for noncompartmental analysis were estimated using Phoenix WinNonlin software version 8.3 (Certara). In noncompartmental analysis at least three time points (median 4, range 3–6) were used to determine the elimination rate constant (kel) from the terminal log‐linear phase of the concentration–time curve. The terminal half‐life (t½) was determined as ln(2)/kel. The linear up logarithmic down method was used in the calculation of AUC (area under the plasma concentration curve). AUC from time zero extrapolated to infinity (AUC_inf_) was reported, as well as the percentage of the extrapolated area within AUC_inf_ (AUC_%Extrap_). Other reported pharmacokinetic values were the plasma CL, the maximum observed plasma concentration (C_max_), the volume of distribution based on the terminal elimination phase (V_z_), and the volume of distribution at steady‐state (V_ss_). Concentrations below the LLOQ were discarded from the analysis. GraphPad Prism version 8 (GraphPad Software) was used for imaging.

## 3 RESULTS

**FIGURE 1:**
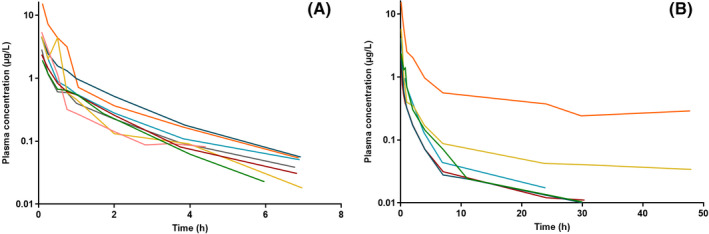
Individual buprenorphine plasma concentrations from (A) eight pregnant sheep that showed quantifiable plasma concentrations up to 7 h after administration and (B) six pregnant sheep that showed quantifiable plasma concentrations up to 48 h after intravenous injection of 10 µg/kg buprenorphine (sheep ID 7 in orange). LLOQ 0.01 µg/L. Concentrations are shown on a semi‐logarithmic scale and are expressed as buprenorphine‐free base

Individual BUP plasma concentration curves are shown in Figure 1, and pharmacokinetic parameters in Table 1, respectively.

**TABLE 1:** Pharmacokinetic parameter values obtained from a noncompartmental analysis of 14 pregnant sheep after intravenous injection of 10 µg/kg of buprenorphine

Parameter	Description	Unit	Parameter value
AUC_inf_	Area under the curve from 0 h to infinity	h*µg/L	2.89 (1.72–40.2)
AUC_%Extrap_	Percent of extrapolated AUC from AUC_inf_	%	4.81 (1.47–27.5)
C_max_	Maximum plasma concentration	µg/L	4.31 (1.93–15.5)
CL	Plasma clearance	L/h/kg	3.39 (0.25–6.02)^a^
t½	Terminal half‐life	h	1.75 (1.07–31.0)
kel	Terminal rate constant	1/h	0.39 (0.022–0.65)
V_z_	Apparent volume of distribution at the terminal phase	L/kg	9.89 (2.91–106.0)^b^
V_ss_	Apparent volume of distribution at steady‐state	L/kg	8.04 (1.05–49.3)

Data are presented as buprenorphine‐free base median (min−max).

^a^ Mean CL 3.40 h*µg/L.

^b^ Mean V_z_ 30.22 L/kg.

Wide between‐subject variability was observed in C_max_ (range 1.93–15.5 µg/L), as well as in other plasma BUP concentrations during the sampling period. Sheep ID 3 and ID 7 showed substantially higher C_max_ values compared to the others (15.3 and 15.5 µg/L vs. 1.93–5.81 µg/L). A post hoc calculation of AUC_0‐7_ (from time zero to 7 h) was done and these values were similar for most sheep (1.49–3.93 h*µg/L) except for sheep ID 3 (8.04 h*µg/L) and ID 7 (15.04 h*µg/L). One sheep (ID 7) showed constantly higher concentrations compared to others throughout the sampling period, as can be seen from Figure 1B. Therefore, sheep ID 7 represents the highest values in AUC_inf_, AUC_%Extrap_, C_max_, and the lowest CL. The reason for this is unknown. For sheep ID 7 the percentage of the extrapolated area in AUC_inf_ was the highest (27.5%) indicating that the parameter estimates for this sheep may be somewhat inaccurate. Interestingly, NBUP was undetected in all samples.

All sheep were sampled up to 48 h, but eight of 14 sheep did not have quantifiable concentrations after 7 h (Figure 1A). In a post hoc analysis it was revealed that those eight sheep that had plasma concentration above the LLOQ only up to 7 h had very similar, short t½ (*n* = 8, median 1.65 h, range 1.07–1.76). When plasma concentrations were quantifiable for 24–48 h, *t*½ were substantially longer (*n* = 6, median 15.2 h, mean 17.6, range 8.16–31.0). The longer half‐life led to lower CL and larger V_z_, as those eight sheep having BUP concentrations above the LLOQ only up to 7 h had a median CL of 3.77 L/h/kg and V_z_ of 9.13 L/kg and for those six sheep having quantifiable concentrations longer 2.85 L/h/kg and 52.2 L/kg, respectively.

## 4 DISCUSSION

The novelty of our study was that, to our knowledge, this is the first pharmacokinetic study of IV BUP in pregnant sheep, and the first sheep study, where blood samples were collected up to 48 h after IV BUP administration. A long sampling period and a highly sensitive quantification method allowed us to obtain more precise pharmacokinetic data during the elimination phase. The use of pregnant sheep was justified as it provided necessary nonclinical data for assessing potential risks to pregnant women and fetuses. Due to ethical reasons, this type of study could not be carried out in humans without prior nonclinical data. High between‐subject variability in pharmacokinetic parameters was observed and originated partly from the fast decline of plasma buprenorphine concentrations below the LLOQ in eight of 14 sheep during the first 7 h after administration. NBUP was undetected in all plasma samples, suggesting that NBUP concentrations are negligible after a single IV dose of BUP 10 µg/kg in sheep.

We conducted the study using 14 sheep, to increase the power of the findings, as high interindividual variability is commonly observed in BUP pharmacokinetic studies.^14^, ^21^ To detect very low concentrations at late time points, we developed a highly sensitive LC/MS/MS method for BUP and NBUP quantification from sheep plasma, that was able to accurately measure BUP concentration at and above 0.01 µg/L with an upper limit of linearity of 25.0 µg/L. In this study, plasma concentrations were collected up to 48 h after IV administration, which proved to be adequate to capture the true elimination phase for those that had measurable plasma concentrations at late time‐points. For these individuals, the terminal half‐life was considerably longer (median 15.2 h) than for those that showed measurable plasma concentrations only up to 7 h after administration (median 1.65 h). In *a post hoc* analysis, we calculated the partial AUC_0‐7_ for all sheep and conclude that the results were similar for most, whereas in the calculation of AUC_inf_ results vary widely. Thus, the longer sampling period unveils a more precise estimate of the between‐subject variability in this study. The long sampling period and the highly sensitive method were key elements for detecting the true elimination pattern of BUP and accurately calculate the pharmacokinetic parameters for IV BUP for pregnant sheep.

Previously Nolan et al., as well as Lindhardt et al. have studied IV BUP pharmacokinetics in nonpregnant sheep.^13^, ^14^ Results from these previous studies have limitations. Lindhardt and associates followed the plasma concentrations only for 1 h, as their study was not designed to investigate the full pharmacokinetic profile of IV BUP in sheep, but the bioavailability of an intranasal formulation. Nolan et al. followed the plasma concentrations for 6 h after IV injection and reported high between‐subject variability in the elimination t½ (mean 2.03 h, range 0.73–5.83). Due to the short sampling period, neither of these studies captured the full terminal elimination phase of BUP. High variability in t½ was also observed in this study, mainly due to a fast decline below LLOQ in many. The sheep that showed plasma concentrations above the LLOQ up to 7 h had t½ close to that observed by Nolan and associates, but t½ in this study increased substantially when able to quantify plasma concentrations for a longer period. High CL for BUP was observed in this study. The mean CL was approximately twice as high as that reported by Nolan et al. in nonpregnant sheep (3.40 vs 1.78 L/h/kg). Higher CL could be explained by the differences in study settings, in sheep characteristics, and/or by the physiological differences between the pregnant and the nonpregnant sheep, such as the increased activity of the metabolizing enzymes and increase in glomerular filtration rate. In human studies, Bastian et al. have shown that BUP exposure (AUC) is approximately 50% lower during pregnancy than postpartum, which compares well with our finding of increased CL in pregnant sheep and indicate the feasibility of our sheep model in BUP pharmacokinetic studies.^9^ From these results, it is evident that a long sampling regimen and a sensitive method are needed to capture the true terminal elimination phase of BUP, and that many of our, and Nolan and associates’, results calculated based on the terminal phase of the concentration‐time curve (AUC_inf_, CL, V_z_, V_ss_) are not that precise due to fast decline below LLOQ. Both previous nonpregnant sheep studies used radioimmunoassay to determine plasma concentrations, which does not discriminate between BUP and NBUP, and the BUP results could be affected by the presence of NBUP. However, in the light of our results, this seems unlikely, as NBUP was undetected in all plasma samples after a single IV injection.

In our pregnant sheep, CL has high between‐subject variability, but generally, the values are much higher (mean 194 L/h for 57 kg sheep) than in nonpregnant human studies. For instance, Huestis and associates observed after 2 mg BUP IV injection mean human CL of 49.8 L/h, t½ of 21.8 h, and V_z_ of 743 L in five male subjects (mean weight 75 kg) sampled up to 72 h.^22^ Previously, Upton reviewed literature and found that the percentage of cardiac output that flows through the liver is higher in (nonpregnant) sheep than in humans (47% and 23%).^23^ The percentages correlate approximately to 155 L/h of liver blood flow in 45 kg sheep and 87 L/h in 69 kg human. This difference could in part explain the higher CL seen in this study, as more blood reaches the liver per unit of time in sheep and can be cleared of the drug that is in the systemic circulation. Notably high CL has been previously observed in an IV pharmacokinetic study done in pregnant sheep for oxycodone in a similar study setting, supporting the observed difference in BUP CL between human and pregnant sheep.^24^ When comparing results from our pregnant sheep noncompartmental analysis to the results from Huestis et al. similarities can be observed for pharmacokinetic values t½ and V_z_. High volumes of distribution were observed for both, human mean V_z_ of 743 L for 75 kg and pregnant sheep mean *V* *_*z*_* 1722 L for 57 kg, respectively. When we were able to detect plasma concentrations for a longer time period, the observed mean t½ is similar to that of Huestis et al., 17.6 h in sheep versus 21.8 h in humans.

The main human metabolite NBUP was undetectable in all sheep plasma samples. Previous studies done by Zullian at al. and Jensen et al. also found no quantifiable concentrations of NBUP in sheep after subcutaneous injection of BUP 50 µg/kg and IV infusion of BUP 40 µg/kg, respectively.^25^, ^26^ In a human study by Huestis et al. IV BUP pharmacokinetic study NBUP was detectable already 10–15 min after injection, and NBUP AUC was on average 18% of that of BUP AUC.^22^ Our data might indicate the lack of BUP biotransformation into NBUP in sheep or highly efficient glucuronidation of NBUP, observed in other animals.^27^

There are limitations in our study. Due to study site logistics, we were unable to perform the study at different stages of the pregnancy, prior to the pregnancy, or postpartum. This would have provided us with a deeper understanding of the effect of pregnancy on the BUP pharmacokinetics in our study population. Additionally, we did not have access to the fetus at the time of the IV study, and thus were unable to determine the fetal exposure of BUP after the injection. The results of this pilot study should be considered preliminary and should be used with caution in a clinical setting. The strength of our study was a highly sensitive analytical method with relatively low LLOQ and a long sampling period that allow us to measure plasma concentration for a longer time period to gain a more precise understanding of the pharmacokinetics of BUP in pregnant sheep.

In conclusion, we uncovered the basic pharmacokinetics of BUP in pregnant sheep after a single IV injection. This knowledge can further be used in consecutive studies in the pregnant sheep model to investigate the transplacental transfer of BUP to the fetus, to increase the knowledge on the safe use of BUP during pregnancy. In the present pharmacokinetic study with 14 pregnant sheep, we have shown that the BUP systemic CL in pregnant sheep is higher than previously reported in nonpregnant sheep and human (male) subjects and that a sensitive analytical method and a long sampling period are the key elements of detecting the true elimination phase of BUP. NBUP, the main metabolite in humans, was undetected in all plasma samples after a single IV injection 10 µg/kg.

## CONFLICT OF INTEREST

The authors declare no conflicts of interest.

## AUTHORS CONTRIBUTIONS

HH, HK, ML, VPR, JR, and MK involved in conception and planning. HH, HK, HMV, and MK involved in data collection. HH, ML, and VPR carried out formal analysis. HH carried out writing the original draft. HH, HK, ML, VPR, JR, HMV, and MK involved in review and editing. HK, ML, VPR, and MK carried out supervision. ML, HMV, and JR involved in the identification of resources.
